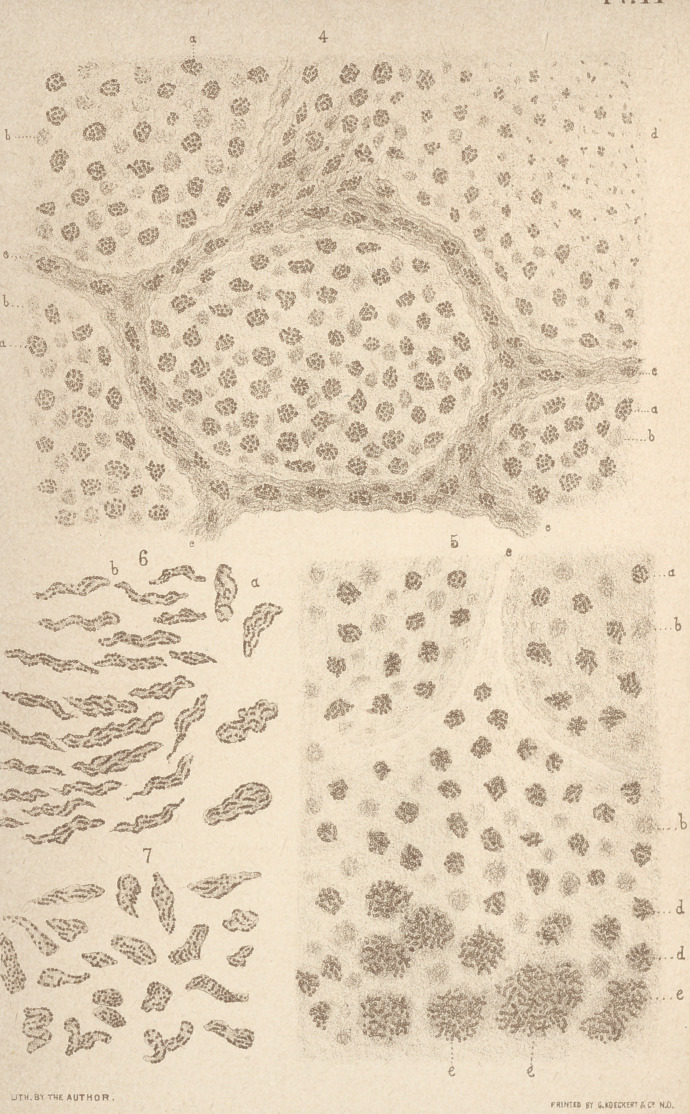# The Origin, Development, Etc., of Tubercle of Tuberculosis

**Published:** 1886-06

**Authors:** H. D. Schmidt

**Affiliations:** Pathologist to the Charity Hospital, New Orleans


THE CHICAGO

### MEDICAL JOURNAL AND EXAMINER.

VOL. LIII. JUNE, 1886. No. 6.

### ORIGINAL COMMUNICATIONS.

IV.

### THE ORIGIN, DEVELOPMENT, DEGENERATION AND DEATH OF THE TUBERCLE OF TUBERCULOSIS. *By* H. D. SCHMIDT, M.D., *Pathologist to the Charity Hospital, New Orleans.*

Although I do not intend to enlarge in this paper upon the pathological anatomy of those organs which, in the human organism, are mostly found to be affected by tuberculosis, some general remarks on this subject are nevertheless demanded, in order to serve as a basis for a thorough discussion on the relationship existing between the bacterium tuberculosis and the particular neoplasm which, in the form of small tubercles, characterize the disease.

The discovery of Villemin of tubercles artificially produced in various organs of certain animals by the inoculation of tuberculous matter, in 1866, and more particularly that of the bacillus tuberculosis by Koch, in 1882, could not but increase the interest which the medical profession has always evinced on the subject of tuberculosis, whilst it gave rise to new, and, perhaps, more thorough investigations into the pathology of this fatal disease, than had been made before. These more recent investigations, including the labors of Villemin and Koch, into the nature and cause of tuberculosis called forth very numerous verbal and written discussions, in which the subject was thoroughly reviewed in all its aspects; so that, at the present time, almost every physician may be familiar with the history of this disease, as well as with the different theories, to which, in the course of time, it had given rise. For this reason I shall forbear making any special remark on this subject, but confine myself to only recalling to the mind of the reader that tuberculosis was formally regarded by many physicians, and still is by some, as a disease distinct from that generally known as pulmonary phthisis, catarrhal or cheesy pneumonia, etc. But although some differences may exist in the course and the accompanying clinical symptoms of these two affections of the lungs, it appears that at the present time they are by the great majority of physicians regarded as identical in their nature. My own investigations into the pathological anatomy of tuberculosis corroborate the correctness of this view, for both affections are from their beginning undoubtedly characterized by the formation of small tubercles identical in origin and structure, though in the one affection these tubercles remain confined to the lungs, whilst in the other they are also met with in other organs. Before continuing, however, our remarks on the identity of cheesy pneumonia and miliary tuberculosis, it appears to me proper to first consider the origin, development, and structure of the so-called miliary tubercle itself, as well as the different changes which it is observed to undergo during the course of the disease in question.

In order to obtain a correct idea of the particular structure and nature of any pathological formation, it is essential that we should have some knowledge of the mode of its origin and development. Such a knowledge is particularly required in the investigation of the miliary tubercle, where the object in view is not limited to its structure and mode of origin, but, moreover, extends to the inquiring into the particular cause by which this neoplasm is called into existence. Accordingly, in commencing these investigations we must direct our attention not only to the smallest, or most recently formed tubercles, but, moreover, to every pathological change, that might be observed to have occurred in the surrounding, apparently healthy, parenchyma. Although the mode of formation of the tubercles is the same in the so-called cheesy pneumonia as in the more typical pulmonary miliary tuberculosis, the most suitable material for the microscopical study of the origin of the tubercles, will nevertheless be found in the lungs, or even other organs, of those cases affected by the last mentioned form of tuberculosis, as here, not unfrequently, portions of the respective organ are met with, in which the disease has not yet passed beyond its incipient stage. Before, however, considering the probable origin and development of the miliary tubercle, let us take a glance at the structural character which it presents when fully developed. The most suitable material for this purpose will be found in the sections, taken from lungs solely affected by miliary tuberculosis, and containing tubercles differing in size, and representing different stages of their growth.

In examining such sections under the microscope it will be seen that the miliary tubercle in the beginning of its formation appears to be limited to one alveolus, whence, however, it soon extends its growth to the adjacent alveoli of the same terminal lobule. It is thus that most of the tubercles, met with in miliary tuberculosis, even the smallest that can be seen by the naked eye, are found to comprise a smaller or larger number of alveoli. In the formation of the larger tubercles, met with in the lungs of cases of cheesy pneumonia, the tubercular growth extends through the interstitial connective tissue to the alveoli of the neighboring terminal lobules. In this manner one of the larger lobules of the lungs, formed by a number of terminal lobules, may eventually be rendered tuberculous. The tubercular growth, however, may even extend beyond this limit and not be arrested until it has invaded a smaller or larger number of the larger lobules, giving rise to one of those tuberculous masses, or nodules, above mentioned. The formation of the larger tubercles or tubercular nodules or masses may here and there be owing to the growth and fusion of adjacent smaller ones, though my own observations have taught me that generally the tubercle of tuberculosis, whether small or large, grows from one centre in a centrifugal direction.

In examining one of the medium-sized or even larger miliary tubercles, contained in a thin section of tuberculous lung, it will be found to be composed of different kinds of cells and other anatomical elements. Whilst its main bulk, including the centre, consists of the regular tubercle cells, averaging in diameter about 1-75 mm., and of an irregular, though epithelioid form, probably produced by their mutual pressure, the cells, observed at its periphery, are smaller in size, resembling rather in form and character the so-called lymphoid or exudation cells. In many tubercles, and imbedded between the regular tubercle-cells, a limited number of cells are met with, distinguished by their unusually large size. These are the so called giant-cells of the tubercle, *apparently* consisting of large masses of protoplasm containing numerous nuclei.

In addition to the cells thus described, however, there is another kind met with, especially in the larger and older tubercles, to which I have already referred in the beginning of this paper. They are best made visible by treating a thin section of tuberculous tissue with a solution of caustic potassa of a strength from 10 to 30 per cent., by the reaction of which all the anatomical elements contained in the section are rendered clear, and eventually destroyed, with the exception of the elastic tissue fibres and these peculiar cells. The latter then appear round or oval in form, mostly larger than the regular epithelioid tubercle cells, and always filled with larger and smaller fat globules. In many tubercles, however, these cells present a dirty brownish or blackish appearance, produced by a smaller or larger number of black pigment granules and minute fat crystals which they contain in addition to the fat globules.

Besides the above-mentioned cellular elements, many of the larger miliary tubercles contain not only the original elastic tissue fibres of the inter-alveolar septa, but, moreover, a connective tissue network, or reticulum, which is regarded by some authors as the stroma of the tubercular neoplasm.

In order to properly understand the relationship in which these different elements, composing the miliary tubercle, stand to one another, it is essential to ascertain the sources from which they are derived. This, however, is a task more difficult to accomplish than one might at first glance imagine. It is for this reason that, though this subject has been quite extensively investigated, some differences of opinion concerning the true origin of the tubercle of tuberculosis still exist among pathological anatomists. The best mode of ascertaining the true sources of these elements consists, of course, as I have already pointed out above, in closely studying the first pathological changes taking place in the intertubercular parenchyma of the lungs, which generally manifest themselves in a multiplication of the cells of one or the other anatomical element. From these primarily formed cells, then, the further development of the tubercle may be pursued and studied in specimens exhibiting the different stages of its growth.

In examining a thin section of miliary tuberculous lung the first signs of the formation of a tubercle may be recognized in a protuberance arising from the wall of one or the other alveolus. From this protuberance the young tubercle is observed to bud, in the form of a little round or conical tumor, into the cavity of the respective alveolus. Generally, the base from which the little tumor arises, exceeds in dimension the tumor itself, but not unfrequently the latter may also be observed to be attached to the wall of the alveolus by a contracted part, or so-called neck. By a continued growth the neoplasm eventually fills up the whole cavity of the alveolus. In most instances, the growth of the base of the tubercle extending over the wall of the alveolus exceeds in activity that of its summit, in consequence of which the tubercle becomes eventually attached to the whole wall of the alveolus, thus completely filling its cavity Not unfrequently, however, though filling up the cavity of the alveolus, it is observed to arise from a smaller base, and with a part of its body detached from the rest of the alveolar wall. In the examination of thin sections of tuberculous lung, not only tubercles partially detached from the wall of the alveolus, but also others, having apparently no connection at all with the latter, are frequently observed. In the latter cases, this entire want of connection between the tubercle and its alveolar wall is easily explained by presuming that during the making of the section the knife passed through the detached portion of the body of the tubercle. As the miliary tubercle grows in a centrifugal direction the particular mode of its origin with the different stages of its development or growth are, of course, best exhibited at the periphery of the tubercle.

But not only in sections taken from lungs of cases of miliary tuberculosis, the particular mode and origin of development of the tubercle is observed to take place in the manner I have described above, for the same is also observed in the growth of those tubercular nodules or masses met with in the lungs of cases of chronic tuberculosis or so-called cheesy pneumonia. In sections taken from such lungs, the young tubercles are likewise seen to arise from the walls of the alveoli, bordering the nodule or tubercular mass, or from those bordering the smaller or even larger bronchioles, where they arise in the form of nipple-shaped buds. In examining attentively the borders of these tubercular masses, some inter-alveolar septa are almost always met with that are distinguished by spindle-shaped enlargements, projecting into their respective adjacent alveolar cavities, and resembling the minute miliary tubercles surrounding the small arteries, or arterioles of the pia mater in tuberculosis of this membrane. In some instances, a tubercular bud is already seen to arise from one side of the enlargement, and to project into its alveolar cavity; in others again, a tubercle is seen to arise upon a moderately extended base from the walls of one or the other alveolus, and to extend into the infundibular cavity which is the more or less filled up by the neoplastic growth. Sometimes, even, I have observed the tubercle to extend through the infundibulum into an adjacent alveolus. When during the cutting of the section of such a tuberculous lung, the knife passes in a transverse, or even slightly oblique direction, through one of these tuberculous polypi, the section of the latter will, of course, be found to lie free in the cavity of its respective alveolus. Thus it happens that in the immediate neighborhood of these tubercular masses sections of small tubercles, distinctly composed of epithelioid cells, and in no way attached to the walls of their alveoli, are not unfrequently met with. As the diameter of these sections of minute tubercles hardly ever reaches that of the alveoli in which they lie, but on the contrary is mostly considerably smaller than the latter—in some instances not more than one-third—there will be always left a smaller or larger empty space between their borders and the alveolar walls. This circumstance, together with their being composed of distinct epithelioid cells, make them resemble minute masses of desquamated epithelium, such as are asserted by some authors to be present in the alveoli of the lungs in cases of the so-called catarrhal pneumonia, the supposed forerunner of pulmonary phthisis. I hesitate not to confess that when I first beheld these bodies I was, myself, inclined to regard them as accumulations of such desquamated epithelial cells, until closer investigation showed me their tubercular origin. In other instances, however, similar minute aggregations of these epithelioid cells may be observed to be attached to the alveolar wall by short and quite narrow pedicles, resembling minute tubercles arising from the epithelium of the alveolus. To these I shall refer again hereafter.

In the foregoing paper I have described the forms in which the miliary tubercles are first seen to arise from the internal surfaces of the walls of the alveoli, but omitted to mention the particular anatomical element directly concerned in their production; in other words, whether they owe their origin to the connective tissue cells of the vessels ramifying between or in the alveolar walls, or may be regarded as a proliferation of the epithelial cells lining the alveoli. As regards this subject, some difference of opinion appears still to exist among pathological anatomists, for while some of them regard the tubercle as a product of inflammation, that is, arising from the cells of some connective tissue, or even from cells migrated from the blood or lymph into the walls of the alveoli, others regard them as a product of the epithelium lining the pulmonary air vesicles. In considering the epithelioid form of these cells, composing the greater part of the miliary tubercle, it appears at first sight difficult to ascertain the true histological origin of the latter. The difficulties met with in the investigation of this subject, however, will be found to diminish and to finally disappear, if our microscopical examinations are sufficiently thorough, and made on a great number of preparations, in order to serve as a reliable basis for sound inductive reasoning. The best way of forming a correct idea of the relationship existing between the different tubercle cells, that is, between those derived from the connective tissue-cells of the vessels, and the others derived from the epithelium of the alveoli, certainly consists in taking into proper consideration their origin in those primary cellular layers of the germ, or blastodermic vesicle, from which the different tissues of the embryo take their start. Let us pursue, therefore, this course, and briefly review the development of the human lungs, more especially that of their parenchyma, such as it is observed in the earliest stages of embryonic life.

The *blastoderma*, or blastodermic vesicle, is formed as we know from those cells resulting from the segmentation of the yelk of the egg, which spread themselves in the form of a single layer over the internal surface of the egg-membrane, the *zona pellucida.* At a certain part of this layer, however, an accumulation of these cells in the form of an hour-glass-shaped disk, representing the so-called "*germinal area*" (*area germinativa*), has taken place, in which the embryo originates. The next phenomenon observed in the development of the latter is the separation of the *blastoderma* into two distinct layers, which may be regarded as the first act of differentiation taking place, for the reason that from the outermost of these layers, called the *ectoderma,* all organs performing the functions of animal life will be formed, while the innermost, the *entoderma*, give origin to all organs destined to perform the functions of vegetative life. Besides this difference of function, however, already at this early period of embryonic life, other differences have been observed to exist, not only in the size, form and general appearance of the cells of these blastodermic layers, but, moreover, in some of their physical properties; for while the cells of the *ectoderma* are small, clear and smooth, and absorb but *feebly* coloring matter, such as carmine, those of the *entoderma* are larger and darker, fewer in number, filled with minute granules, and absorbing the carmine quite intensely; besides, they are rendered darker by the reaction to osmic acid than the ectoderm-cells on account of the minute fat-globules which they contain. This difference of degree in the absorption of coloring matter, or in the reaction to osmic acid is, according to *Haeckel*, already observed in the first pairs of cells, resulting from the cleavage of the ovum.* As soon as the first morphological changes, indicating the formation of the embryo, take place in these germinal layers, another layer appears between them. This is the *mesoderma*, or so-called " motor and germinative " layer, from which all the parts of the body consisting of connective tissue, muscles and nerves, together with the urinary and generative organs are formed. During the primary stage of development of the embryo, however, the *mesoderma*, also, splits up into two subordinate layers, the outermost of which, the *lamina inodermalis* (Haeckel) is destined for the formation of the corium, muscles of the trunk and extremities, and the bones, while the innermost, the *lamina inogastralis* (Haeckel) gives origin to the muscles and fibrous coats of the intestines and their glands, the muscles of the pharynx, æsophagus, stomach and other appendages of the alimentary canals, as well as to the heart and the most important blood vessels. The divisions of the *mesoderma* may be regarded as another act of differentiation concerning the functions of those organs for which its two layers are destined, *i. e.*, the one for organs of animal, the other for organs of vegetative life. Thus the original *blastoderma* now consists of four distinct layers, which, counted from the outside, have been denominated by Haeckel as follows: 1. *Lamina neurodermalis* (*ectoderma*); 2. *Lamina inodermalis*; 3. *Lamina inogastralis* (*mesoderma*); 4. *Lamina mycogastralis* (*entoderma*).

As regards the origin of the *mesoderma*, however, differ-

* H. E. Haeckel.—"*Anthropogenie oder Entwicklungsgeschichte des Menschen.*" 3rd edition, p. 172.

ences of opinion exist among the different investigators of this subject, and the question is, as yet, not decided, whether the middle layer is derived from *ecto-* or *entoderma*, or whether its subordinate layers may not be derived from both, that is, the outermost from the *ectoderma*, and the innermost from the *entoderma*. For certain reasons, I am inclined to give preference to the statements and views of A. Koelliker,* one of the oldest and most reliable investigators, who regards the *mesoderma* as being derived from the *ectoderma* alone.

Having by the above review of the formation of the primary cellular layers of the embryo now obtained a basis upon which to rest our arguments concerning the origin and signification of the different kinds of cells of which the miliary tubercule is composed, we may turn our attention to the origin and development of the lungs, representing those organs in which the tubercle is most frequently met with. In the embryo the lungs are found to originate in the upper part of its primary alimentary canal, called the "foregut," where they first appear in the form of a minute dilatation, which, by gradually lengthening, assumes the form of a tube which, in its turn, gives rise to two similar tubular processes. While the latter represent the primary larger bronchi, the parent tube represents the trachea. From each of these primary bronchi, then, two other subordinate bronchi originate which, again, in their turn, give rise to other subordinate branches, until, by the continued formation of new and smaller ones, the whole bronchial system is eventually developed. The primary alimentary canal, from which the trachea and bronchi take their origin, is composed, as we know, of two blastodermic layers, viz.: the *entoderma*, and the inner division of the *mesoderma*, the

* A. Koelliker.—"*Entwicklungsgeschichte des Menschen und der hoeheren Thiere.*" 2d edition, p. 268.

*lamina inogastralis* of which the former is represented by the epithelium and the latter by the fibrous envelope of the canal. These same layers, of course, build up the trachea and bronchi. According to Koelliker, however, in the beginning of the development of the bronchial system, the *entoderma* alone takes part in the formation of the bronchial branches, which bud, so to say, into the surrounding cellular mass of the *lamina inogastralis.* This phenomenon seems to indicate that the development of the bronchial system mainly proceeds from the *entoderma.* Soon after, of course, the *lamina inogastralis* also takes part in the process of formation of the bronchial ramifications, so that each new bud, arising from the end of its predecessor, consists already of two membranes ; though, even now, the epithelium of the bronchial tubes still takes the lead in the formation of new buds. By the successive formation of new subordinate bronchial tubules the whole bronchial system appears developed at the sixth month. During the development of the latter the newly formed bronchial tubules present at their ends spherical dilatations, designated by Koelliker "*primitive gland-vesicles*," and from which, in turn, the new bronchial buds arise, but which as yet do not represent the pulmonary air vesicles or alveoli of the fully developed lungs. Already in the fourth month, Koelliker found all the bronchial tubules, with the exception of their vesicular terminations, lined by a ciliated epithelium. In the sixth month, finally, the alveoli are formed in the manner that the vesicular buds, arising from the last-formed primitive gland-vesicles do not separate any more from one another during their growth into pedunculated vesicles, but remain connected to one another, opening besides into a common space which represents the so-called "infundibulum." These terminal vesicles are, furthermore, distinguished by a flat pavement-like epithelium.

The above sketch which I have drawn of the development of the lungs in the earlier stages of embryonic life, shows that, while the greater part of these organs, comprising the fibrous and muscular elements of the larger and smaller bronchi, together with the walls of the alveoli, as well as the larger and smaller blood vessels, are derived from the *mesoderma*, which itself is a product of the original " animal layer " of the blastodermic vesicle, the *ectoderma*—the remaining part consisting only of the epithelia of the bronchi and alveoli may be traced back to the " vegetative layer," the *entoderma*. But as the miliary tubercle is found to be built up chiefly by comparatively large cells, epithelioid in form, it would almost appear to be a product of the epithelial cells lining the alveoli. This, however, is not the case, for, though these tubercle cells do, to a certain extent, resemble in form those of an epithelium, they, nevertheless, as we shall see directly, are derived from another source. But even if we should fail to trace them back to another tissue than the alveolar epithelium, we might point to a certain phenomenon, observed in the life history of the miliary tubercle, which clearly shows that this neoplasm can not be the product of an epithelium. I refer to the phenomenon observed in those cases of so-called " fibrous miliary tubercles," in which the tubercle cells become organized into a tissue of a higher grade, into a sort of connective, or so-called " cicatricial tissue." This process of organization, as we know, is never observed to take place in the cells of an epithelium, or in those of a gland, but, on the contrary, always involves the cells of connective tissues, or their descendants. Nor is it limited to cases of fibrous tubercles, for, in those indurated portions of lungs of cases of chronic tuberculosis, also, tubercle cells may be seen to elongate and to be converted into a ort of coarse connective tissue.

The best example, however, for showing that the miliary tubercle is not a product of an epithelium, are those tubercles met with in the peritoneal layers of the mesentery and intestines, but still more those which are formed around the small blood vessels of the pia mater. More than four years ago, I made a number of examinations of the tubercles surrounding the blood vessels of this membrane, and distinctly saw them arise from the connective tissue cells of their adventitia. Some of these vessels were so minute, that, though they were stained with picro-carmine, it was difficult to distinguish them by the unaided eye. Suspecting at first the tubercles to arise from the endothelium of these vessels, I directed my special attention to this layer, the cells of which were easily distinguished in the smaller arterioles, but failed in my expectations, as I found, in every instance examined, the endothelium entirely free from tubercle cells, while in the adventitia they were met with in all stages of development. Arising from the cells of the adventitia of the arterioles, or even from the nuclei of the capillary vessels—which generally are also surrounded by a thin layer of cell protoplasm—the tubercle cells multiply by division, and form tubercles too minute to be seen by the naked eye. Though of a moderate diameter, when first arising from the connective tissue cells of the adventitia, they soon enlarge, until they have attained the diameter of those cells, composing a fully developed tubercle, while by mutual pressure they are rendered polygonal in shape resembling then the cells of an epithelium. It must not be overlooked, however, that there are many tubercle-cells, which present very irregular forms, without resembling in any point a regular epithelial cell. The minute tubercles, when first formed, are observed to rest upon the wall of the vessel in the form of minute irregularly shaped masses, though by continued growth they finally surround it, and assume that ovoid form characteristic of the tubercles of these vessels. The diameter of these tubercles, when fully grown, are generally proportionate to those of the individual vessels upon which they are found. The miliary tubercles met with in the peritoneal layers of the mesentery must, as there exists no epithelium in their close vicinity, of course, arise from the cells of some connective tissue, or, perhaps, as Rindfleisch states, from the endothelium of the lymphatics, or blood vessels. As regards the tubercles of the small intestines I may state, that I have endeavored to trace the connection of these tubercles by carefully separating under water, and with the aid of a strong loupe, the different layers of the intestines. In a number of instances I found the little tumor arising from the fibrous, and extending through the muscular coat. In others, however, as may also be seen in thin vertical sections of the intestines passing through the tubercle, the latter involved the fibrous layer of the mucous membrane where it may finally by its degeneration give rise to tuberculous ulceration. In examining a small miliary tubercle of the liver, the tubercle cells were also seen to arise from the cells of the minute blood vessels, though I would, for the present, not positively assert, that the hepatic cells take no active part in the formation of the tubercle. The same appears to be the case in the kidneys, though in these organs I have, in some instances, met with glomreruli, the vessels and also the capsules which presented the appearance of having been transformed into a sort of connective tissue.

In studying the mode of the peripheral growth and extension of the tubercles in thin sections of tuberculous lung, it will be observed that it always takes place along the adjacent alveolar septa, which are thickened by the multiplication of of smaller or larger tubercle cells arising from the connective tissue cells of the adventitia, or, perhaps also, from the cells of the endothelium of the vessels, ramifying in these septa. It is thus that in these sections the outlines of the tubercles, when examined under the microscope, appear but rarely as evenly curved lines, though to the naked eye the little round or oval tumors may present a smooth appearance. In most instances, the borders of the miliary tubercles are irregular, to a certain extent star-shaped, or multipolar, the processes being formed by the adjacent inter-alveolar septa, along which the growth of the tubercle proceeds. This is especially the case in those large tubercles and tuberculous masses, met with in those lungs affected with chronic tuberculosis, in which the mode of extension of the tubercular process may be studied to advantage.

After having in the preceding paragraphs endeavored to show that the tubercle cells are most probably derived from the connective tissue cells of the adventitia of the vessels, ramifying in the inter-alveolar septa of the lungs, I shall now pass over to a brief consideration of the general character and relations of all the cells met with in the anatomical composition of the miliary tubercle. Presuming, then, from what has been said above, that the true tubercle-cells are descendants of connective tissue cells, it still remains for us to determine the origin of the other kinds found in their company. In considering first the origin and signification of those small lymphoid cells, generally met with along the periphery of the tubercle, we may suggest that they represent true exudation cells, or, in other words, colorless blood corpuscles, emigrated from the neighboring congested blood-vessels; or, as they are not met with in the main body of the tubercle, they might also be regarded as young tubercle-cells, derived, like the others, from connective-tissue cells. In most instances, the neighboring blood-vessels of the miliary tubercles are found congested, and, if this is not the case, it may safely be presumed that they were congested during, and even previous to the formation of the tubercles; for it can hardly be supposed that the tuberculous process would take place and continue without an increased activity of the minute blood-vessels, giving rise to a morbid irritation of the connective tissue cells of their adventitia. The abnormal activity of these vessels, however, would soon be followed by a relaxation of their walls, causing a retardation of the blood-current within them, which would finally lead to a permanent congestion of these vessels. Under these circumstances it is not improbable that a number of colorless blood corpuscles emigrate from the blood-vessels, and in association with the tubercle cells—which at the same time are formed from the irritated and now multiplying connective tissue cells of the adventitia—take their part in the formation of the tubercle. It is thus that these lymphoid cells are always met with at the periphery of the tubercle, or in the alveolar septa.

The next kind of cells to be considered are the so-called "giant cells" which, greatly differing among themselves in their diameter, and individually containing a smaller or larger number of nuclei, are principally met with in the older and larger tubercles. Although surrounded by the general mass of tubercle cells they remain unconnected with the latter. In thin sections of tubercles, they appear finely granular, mostly round or oval, though sometimes irregular in form; they are surrounded by an empty space which separates them from the regular tubercle cells, though, in some instances, they appear connected to the latter by processes arising from their body. Although these giant-cells have frequently been the subject of special study, pathological anatomists still differ in their views as to the origin, nature and signification of these formations. By some they have been regarded as representing coagula within the bloodvessels, around which the tubercle is developed, their nuclei being partly derived from the colorless blood corpuscles of the coagulum, partly from the endothelial cells of the vessels; or, also, the whole giant cell representing a conglomeration of colorless blood-corpuscles;—while on the other hand they have been looked upon as conglomerations of epithelioid tubercle cells, or of epithelial alveolar cells. Again, according to another view, they are derived from the connective tissue cells, or also, from the endothelial cells of the lymph-spaces. When met with in tubercles of the testicle, liver, kidney, and mammary gland, they have been regarded as derived from the epithelia of the seminiferous tubules, hepatic ducts, uriniferous tubules, and milk ducts. Without enlarging upon the various theories existing on the origin of these giant cells, we may say, that they are generally considered to represent smaller or larger masses of protoplasm, derived either from the growth of one cell, the nucleus of which alone was subjected to the process of multiplication, or from a fusion of the protoplasm of a number of cells with single nuclei. From my own observations it appears to me that perhaps the greatest number of these giant cells are formed from coagula of blood within the blood-vessels. I have been convinced of this by observing, especially in thin sections of fibrous tubercles, a number of these cells or masses of protoplasm still to consist of a mass of brownish polygonal cells, lying loosely in, or even connected by some points of their periphery with the wall of empty spaces, representing most probably the lumina of blood-vessels. The walls of the latter, though fused with the surrounding tubercle, and, like this, consisting of long, spindle-formed cells, could be still distinguished. Whether the polygonal cells, generally surrounded by a brownish, finely granular, substance, were derived from the colorless blood-corpuscles of the coagula, or from the cells of the endothelia of the vessels, I leave for the present undecided. Generally, there were no colored blood-corpuscles observed in the mass, a circumstance, which is, however, easily explained by assuming that the protoplasm of these corpuscles had been appropriated by the other cells to serve them as nourishment during their growth, while the hæmoglobin of the former was left behind. The degeneration of the protoplasm of the giant cell thus formed, which, of course, eventually takes place, causes the obliteration of the outlines of its component cells, so that the whole formation assumes a finely granular brownish appearance. A number of giant cells presenting this appearance and containing a smaller or greater number of nuclei are, therefore, always met with in the tubercles of the same section of lung. But though, as I have said above, the greater number of giant cells may be formed in the manner just stated, I have good reasons to believe that others represent conglomerations of cells of the alveolar epithelium, which, during the growth of the tubercle, were surrounded by the multiplying cells of the latter. To this view I was chiefly led by meeting in a number of alveoli with certain conglomererations of cells, which, presenting the average size of a giant cell, resembled those cells lining the wall of the alveolus, to which they appeared to be attached. These giant cells do not present a brownish appearance, but absorb coloring matter more intensely than the surrounding tubercle cells. I have never observed giant cells converted, like the true tubercle cells, into any form of connective tissue.

Besides the giant cells there is another kind of cells met with in the miliary tubercle, the origin of which we shall now consider. I refer to those peculiar cells, which are characterized by their brownish or blackish appearance, and by the fat globules and fat crystals they contain; they are, as before mentioned, met with in sections of older tubercles, especially when treated with a solution of caustic potassa. These cells are generally single, and mostly met with along the periphery of the tubercle. They always contain a considerable number of smaller and larger fat globules, and many of them fat crystals, and black pigment granules besides; the latter, in fact, causes their brownish or blackish appearance. By these characters they are easily distinguished from true tubercle cells; for, though many of the latter also undergo fatty degeneration, they do not contain so great a number of large fat globules as the former, nor do they contain fat crystals, or pigment granules. I doubt not but that these cells are identical with the epithelial cells lining the alveolar cavities which, during the growth of the tubercle, are surrounded by, and inclosed in the mass of growing tubercle cells, to finally undergo the fatty metamorphosis, whilst in the older tubercles the fat globules will be converted into fat crystals, or, even, into black pigment. Not unfrequently these cells are also met with in the expectoration of chronic cases of tuberculosis.

There is another anatomical element met with in many, though not in all tubercles, which like the giant-cells has frequently been a subject of discussion, or even of controversy, among different investigators. This is the so-called "reticulum." As far as my own observations extend, I may state that such a reticulum, which by some authors has been compared to the stroma of some pathological tumors, appears to exist in many miliary tubercles, especially in the older ones, whilst in others it is certainly absent. For the reason, however, that I entertain some special views on the origin of this reticulum, which to explain would consume too much space in this treatise, I must for the present forbear to enlarge upon this subject and postpone its discussion to some other occasion.

In the great majority of cases of tuberculosis, the miliary tubercles or tuberculous masses undergo a certain kind of degeneration, known as "coagulation necrosis" which lead to their final destruction. Not unfrequently, however, the cells of the miliary tubercle, instead of undergoing this retrograde metamorphosis, show an inclination to become organized into a higher grade of tissue, by gradually assuming a more and more elongated form, in order to be finally converted into a sort of connective, or so-called "cicatricial tissue." This process may be observed in all its various stages in those indurated portions of lung met with in cases of chronic tuberculosis, where it involves not only the tubercle cells, but also those of the interstitial connective tissue of the organ as well as of the blood-vessels, etc. The tendency of the tubercle-cells toward organization is, moreover, and perhaps more distinctly, displayed in those cases of so-called "fibrous tubercle," the lungs of five of which I have microscopically investigated Here, the tubercles, though miliary in character and mostly small in size, are frequently found lying very near to one another, forming smaller and larger groups or masses. Sometimes even, they fuse with another, though it can always be seen that each tubercle had developed from its own individual center. These tubercular groups, or masses, I mostly found in the apices of the lungs; only in some instances I observed them, as also single fibrous tubercles, in the other lobes of these organs. In all cases of fibrous miliary tuberculosis that came under my notice, the tubercles were not what might be called "numerous" as we often observe in acute miliary tuberculosis, but they were almost always associated with a greater or less peri-bronchitis fibrosa, or with interstitial pneumonia (cirrhosis of the lungs), and generally characterized by an abundant formation of black pigment.

It is not often, however, that the tubercular process in the lungs is arrested by a fibrous metamorposis of the tubercle cells; on the contrary, in the great majority of cases of tuberculosis, so already mentioned, the disease is carried to a fatal issue by a retrograde metamorphosis, the so-called "cheesy degeneration," or "coagulation necrosis," which the elements of the tubercular growth gradually undergo. By this process, which always commences in the center of the tubercle, and progresses toward its periphery, the protoplasm of the tubercle-cells appears to coagulate and to be converted into a cheesy substance, by the final disintegration and liquefaction of which a cavity is formed in the center of the tubercle. In the course of the degenerative process the outlines of these cells become obliterated, and their protoplasm is gradually converted into a cheesy granular mass, containing minute shapeless bodies or particles. These bodies, I suppose, represent to a certain extent the remains of the nuclei of the tubercle cells, which appear to possess a greater power of resistance to the pathological cause of the process than the protoplasm of the cells. The direct cause of the coagulation-necrosis of the miliary tubercle has, as yet, not been positively determined, though the degeneration is commonly ascribed to a want of nutrition due to the absence of blood-vessels in the tubercle. In examining thin sections of tuberculous tissues we already observe traces of the degenerative process in almost all tubercles; it is only the smaller ones, or those most recently formed, that are still free from degeneration. In the older and larger tubercles, small cavities, formed by the final disintegration of the cheesy mass, are observed in their center, whilst at the periphery the tubercular growth is still going on. In those large tubercles, formed by the meeting and fusion of a number of smaller ones, several centers of degeneration and disintegration may exist at the same time. By the breaking down of these tubercles a number of small cavities are formed throughout the tuberculous portion of the lung, which, in the course of the disease, may enlarge by the ulceration and final destruction of the intervening non-tuberculous parenchyma. Such a condition is mostly observed in miliary tuberculosis. In some cases of chronic tuberculosis, however, smaller or larger circumscribed cheesy masses, surrounded by healthy parenchyma, are frequently met with, in which only one cavity, produced by the breaking down of their substance is found to exist. From this circumstance it appears that in these masses, some of which are more or less spherical in form, the tubercular process started from only one center, and after having proceeded in a radiating direction to a certain extent, was by one or the other cause arrested. These formations represent, in truth, miliary tubercles on a large scale. They are, generally, partially detached, partially still connected with the surrounding healthy parenchyma. Several years ago, I have hardened quite a number of these circumscribed cheesy masses, or nodules, some of which as large as a walnut (about one and a quarter inch in diameter), in Mueller's fluid, followed by alcohol, with the view of investigating their exact nature in thin sections. The microscopical examination of these sections showed me that, while at the borders of the cavities the substance of these giant tubercles was, as in the ordinary miliary tubercles, completely degenerated and disintegrating, their former structure could be still distinguished throughout the greater part of the section. Not only the outlines of the numerous alveoli, bronchioles, interstitial connective tissue, etc., which had been involved by the growth, but also, toward the periphery of the latter, those of the tubercle cells could still be recognized. The borders of these circumscribed masses were formed by long structureless bands, concentrically arranged around their periphery and connected with the surrounding non-tuberculous parenchyma. No traces of the outlines of elongated cells, indicating their formation from tubercle-cells, could be discovered in these bands; but though of a smooth homogeneous aspect, they rather appeared to have at one time represented bundles of neoplastic connective tissue, the product of some inflammatory process. A closer study of the subject subsequently showed me that they represented the former walls of the adjacent alveoli of the surrounding non-tuberculous parenchyma, which, being put on the stretch by the increasing dimensions of the tuberculous growth, had adapted themselves to the peripheral form of the latter, and, through the inflammatory irritation extending from the neighboring tuberculous mass to their blood-vessels, were eventually converted into bundles of a coarse neoplastic connective tissue. That this was the true explanation of the phenomenon observed, was proved by the surrounding alveolar walls of the healthy parenchyma, which, still connected to these fibrous bands, presented in the section the form of more or less elongated meshes, produced by the stretching of the lung parenchyma.

The conversion of the alveolar walls into neoplastic connective tissue, however, is not only observed around the periphery of the above described tuberculous masses, but takes place also in other localities of tuberculous lungs, for instance, along the borders of those portions of indurated lung, met with in chronic tuberculosis. Even, along the periphery of miliary tubercles, sections of the alveolar walls of the surrounding parenchyma are frequently observed to present the form of elongated meshes. A similar aspect is presented to the eye of the observer in the microscopical examination of sections made from lungs affected with peribronchitis fibrosa. Here sections of obstructed bronchial tubes will be met with in which the original structure of their walls is entirely obliterated, and the whole tubule appears to be converted into connective tissue; the cavity seen in the center of the tubule represents the disintegrating fibrinous thrombus. The sections of such bronchioles somewhat resemble those of miliary tubercles, though a closer examination will show no trace of a tubercle cell, but rather that they consist of concentrically arranged homogeneous looking bands, some of which are still connected with the elongated alveolar walls of the neighboring parenchyma of the lungs.

Although the formation of these fibrous bands along the borders of tubercles, otherwise tuberculous portions of lungs, might be regarded as a means of nature to arrest the morbid process of tuberculosis, this is rarely the case. On the contrary, the homogenous structureless appearance of this neoplastic connective tissue already shows that it will be, like the tuberculous portions of lung which it borders, finally subjected to the coagulation necrosis. The elongated spindle shaped tubercle-cells, playing a prominent part in the process of induration of certain portions of tuberculous lung, either such as they are, or transformed into a coarse connective tissue, are in most instances likewise doomed to undergo the same destructive process. It is this that in cases of chronic tuberculosis very considerable portions of lung are frequently destroyed and large cavities formed. The only organs, capable of resisting this general destruction, are the larger blood vessels and bronchial tubes, which are frequently met with stretched across these cavities in the form of single cords. Like the ordinary miliary tubercle, the fibrous tubercle, also, is subject to undergo, at least in its center, the coagulation necrosis.

From the above sketch of the origin, development, retrogression and death of the miliary tubercle in its various forms, it will be seen that the tubercular process is very complicated, and that it closely resembles in its products and their metamorphoses the process of chronic inflammation. To convince ourselves of the truth of this assertion we have but to examine and study the condition of the lungs of a case of chronic tuberculosis. In such lungs all the various stages of the inflammatory process will be found represented in different localities of these organs. Thus we may find the superior lobe hollowed by smaller or larger cavities, which, frequently communicating with one another, are like a chronic abscess lined by a so-called pyo-genetic membrane, and contain a greater or lesser quantity of purulent matter. The remaining parenchyma of the lung between these cavities is generally found indurated, and, when miscroscopically examined, presents to the eye of the observer a most variegated picture of its anatomical elements passing through the various stages of the inflammatory process. Approaching in our examination the base of one or the other lung we may find, according to the prevailing circumstances of the case, a decrease of the inflammatory destruction in the absence of the cavities, whilst the parenchyma, though still congested and containing large tubercles or tubercular masses, appears more soft and spongy than in the upper portion of the lung ; or, at the very base of the organ, the parenchyma may even present the appearance of a typical miliary tuberculosis. In accordance with the special conditions of the different parts of such a lung; its surface also presents a variety of colors and their shades, for, whilst one portion of it may appear more or less gray by the induration of its parenchyma or the thickening of the pleura, another portion may by the congestion of its vessels present a red or blue appearance, while a third will exhibit all the macroscopical characters of emphysema. Thus it happens that, in severe cases of chronic tuberculosis, a combination of otherwise individual inflammatory diseases of these organs, such as bronchitis, peribronchitis, bronchiectasis, interstitial pneumonia, etc., may be found to exist at one and the same time.

With the foregoing paragraph I close the general remarks on the pathological anatomy of tuberculosis, to which I referred in the beginning of this division of the present treatise. The sketch which I have drawn of the origin, development and death of the tubercle of tuberculosis is, though it may serve my present purpose, far from being complete. But as the reader may infer, from what I have already said above, the subject of tuberculosis is too complicated and extensive for a thorough discussion in all its details in the limited space of my present treatise, requiring rather the numerous pages of a whole volume. Whatever, therefore remains to be said on the pathology of tuberculosis I will have to postpone to a more convenient occasion.

## Figures and Tables

**Figure f1:**